# Diagnostic Methods for Bovine Coronavirus: A Review of Recent Advancements and Challenges

**DOI:** 10.3390/v17121533

**Published:** 2025-11-22

**Authors:** Jie Dong, Xiaoxiao He, Shijun Bao, Zhanyong Wei

**Affiliations:** 1 College of Veterinary Medicine, Gansu Agricultural University, Lanzhou 730070, China; 1987368201@163.com (J.D.); 15117161449@163.com (X.H.); 2 College of Veterinary Medicine, Henan Agricultural University, Zhengzhou 450002, China

**Keywords:** BCoV, moleculardiagnostic methods, CRISPR/Cas, immunological methods, lateral flow assay (LFA), integrated platforms

## Abstract

Bovine coronavirus(BCoV) is a significant pathogen causing substantial economic losses in the cattle industry through increased calf mortality, reduced growth performance, and decreased milk yield. Rapid and accurate diagnostic methods are therefore essential for controlling BCoV transmission. Current diagnostic methods comprise two primary categories: conventional techniques and cutting-edge innovations. Conventional approaches, including molecular methods like RT-PCR/qRT-PCR and immunological assays such as ELISA and neutralization tests, remain the main diagnostic methods. However, they are limited by laboratory dependency as well as the necessary balance between speed and sensitivity. These limitations have promoted the development of innovative methods, including isothermal amplification, CRISPR/Cas systems, droplet digital PCR, and integrated platforms. This review comprehensively analyzes the advantages, limitations, and applications of current diagnostic methods, highlighting integrated platforms such as RPA-CRISPR-LFA and microfluidics-based LFA. These innovations bridge critical performance gaps by enhancing sensitivity and specificity while enabling field application, demonstrating significant potential as next-generation point-of-care diagnostics for managing this economically critical pathogen.

## 1. Introduction

Bovine coronavirus (BCoV), first identified in 1973, is an enveloped positive-sense RNA virus with a global distribution [1,2,3,4]. BCoVis primarily transmitted through the fecal-oral routes, aerosolsand respiratory droplets, with persistent carriers leading to herd-level outbreaks [5,6,7]. BCoV infection manifests three distinct clinical syndromes: high-mortality calf diarrhea (CD), winter dysentery (WD) in adult cattle, and bovine respiratory disease complex (BRDC) [8,9,10]. These infections cause substantial economic losses through elevated calf mortality, reduced feedlot performance, and milk yield reductions with reductions reaching 70% during WD outbreaks [11,12,13,14]. BCoV has broad host tropism, including elk, deer, and camels [15,16]. The virus’s broad host tropism further complicates disease surveillance and control. This review will focus on the evolution of diagnostic methods designed to address these complex challenges.

Despite the international availability of inactivated and live attenuated vaccines, their production complexity, cold-chain dependency, and regulatory delays collectively increase breeding costs and limit widespread adoption in many regions [17]. Consequently, rapid and accurate diagnostics remain critical for effective BCoV control. Conventional diagnostic methods, such as virus isolation and electron microscopy observation, remain restrained by their dependence on sophisticated equipment, specialized personnel, and centralized laboratory settings, resulting in prolonged detection times [18,19,20,21]. Current BCoV diagnostic approaches fall primarily into two categories: nucleic acid-based molecular diagnostics for detecting viral genetic material, and immunological diagnostics for identifying viral antigens or antibodies. While significant progress has enabled rapid field diagnostics for on-site detection, multiple challenges still hinder their widespread deployment.

This article comprehensively reviews recent advances in BCoV detection methods and analyzes their potential for field implementation in resource-limited settings. By critically assessing the advantages and limitations of each technology, this review aims to guide the development of accurate, user-friendly, and rapid point-of-care tools for BCoV surveillance and outbreak management.

## 2. BCoVGenome Structure andIsolation Characteristics

**Figure 1:**
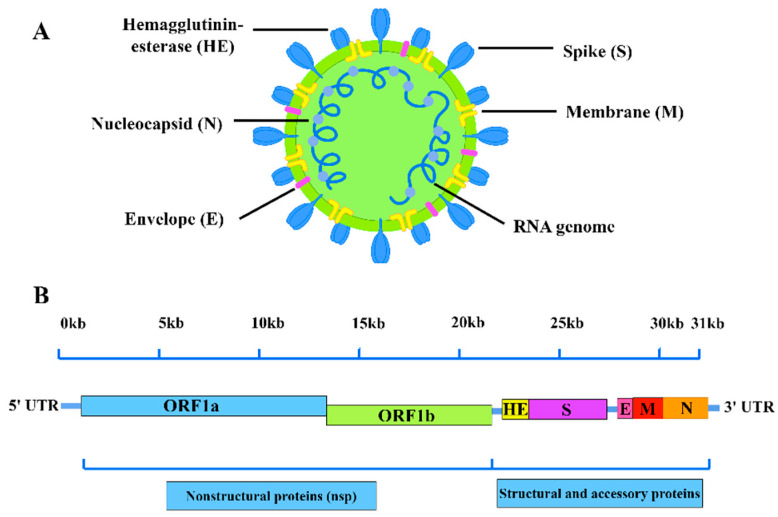
Schematic representation of the BCoV virion structure (**A**) and genome (**B**) (By Figdraw).

BCoV belongs to the order *Nidovirales*, family *Coronaviridae*, genus *Betacoronavirus*, and species *Betacoronavirus 1* [22]. Its genome consists of a single-stranded RNA (~31 kb) flanked by a 5′ cap and 3′ polyadenylated tail, containing 13 open reading frames (ORFs) [23]. The 5′-terminal two-thirds encode ORF1a/ORF1b, which are processed into RNA-dependent RNA polymerase and non-structural proteins. The 3′-terminal region encodes five major structural proteins-spike (S)protein, membrane (M)protein, hemagglutinin esterase (HE)protein, envelope (E) protein and nucleocapsid (N)protein (Figure 1) [17]. The N protein, highly conserved among BCoV strains, serves as a primary target for molecular diagnostics. The S protein, composed of the receptor-binding S1 subunit and the membrane fusion S2 subunit, induces neutralizing antibodies and is ideal for immunological diagnostics [24,25].

Virus isolation remains a foundational technique for confirming active BCoV infection and propagating the virus for further characterization. BCoV is shed in feces and nasal secretions from both symptomatic and asymptomatic cattle. Virus particles are typically isolated from fecal samples using human rectal tumor-18 (HRT-18) cells [19,26]. Successful infection is usually evident within a few days by the appearance of a cytopathic effect (CPE), characterized by dense clusters of enlarged, rounded cells and syncytia, culminating in cell lysis. Viral replication is further confirmed by techniques such as immunofluorescence assay (IFA), RT-qPCR, or electron microscopy. Although frequently detected in healthy cattle [27], BCoV’s clinical impact is significantly amplified in co-infections. Epidemiological studies demonstrate that interactions with enteric pathogens such as *Escherichia coli*, rotavirus or respiratory viruses, including bovine respiratory syncytial virus (BRSV), parainfluenza virus-3 (BPIV-3), exacerbate disease severity and mortality, particularly in calves [28,29,30,31]. High co-infection rates in both symptomatic and asymptomatic animals, combined with challenges in isolating BCoV from conventional cell cultures, complicate final diagnosis [32]. Modern molecular techniques such as RT-PCR have largely replaced traditional culture-based methods, enabling sensitive and specific detection of BCoV and co-infecting pathogens. These advancements are critical for improving surveillance and outbreak control, despite persistent difficulties in viral isolation [19,25]. Consequently, virus isolation is now largely confined to pathogen characterization rather than clinical diagnostics.

## 3. Molecular Methods for Viral RNA Detection

Molecular detection remains the primary diagnostic method for BCoV. These nucleic acid-based methods demonstrate higher sensitivity and specificity in detecting viral RNA sequences, enabling earlier pathogen identification compared to antigen- or antibody-based immunological approaches. Currently, the primary molecular diagnostic methods employed for BCoV detection include reverse transcription polymerase chain reaction (RT-PCR), real-time RT-PCR, droplet digital RT-PCR, isothermal amplification, next-generation sequencing and clustered regularly interspaced short palindromic repeat systems.

### 3.1. Reverse Transcription Polymerase Chain Reaction (RT-PCR)

RT-PCR targeting specific BCoV RNA fragments is now a widely adopted diagnostic method due to its superior specificity and sensitivity compare to immunological approaches. This performance is achieved through the use of well-designed primer sequences that bind specifically to a unique target site within the organism’s genome, facilitating selective amplification and detection. The most frequently targeted genomic regions include the N, Sand ORF1ab genes [33].

In previous report, an RT-PCR assay targeting a 407 bp fragment of the BCoV N gene was developed to detect BCoV in fecal samples from experimentally inoculated cattle [34]. This RT-PCR assay showed high sensitivity and specificity when validated against tissue culture-adapted BCoV strains and feces from inoculated calves. In this study, ten BCoV-seropositive adult dairy cows were inoculated intranasally, orally, or via surgically implanted duodenal catheters with BCoV. Mild diarrhea developed in six cows inoculated through intranasal or duodenal routes, with RT-PCR confirming BCoV existing in diarrheic feces. These results highlight RT-PCR’s reliability as a diagnostic tool for BCoV detection in both calves and adult cattle. Building on this foundation, other researchers developed an RT-PCR assay for detecting BCoV in nasal swabs and fecal samples from experimentally infected calves [35]. Following inoculation, all calves developed diarrhea, and BCoV’s RNA was consistently detected in diarrheic fecal samples and corresponding nasal swabs. The assay demonstrated high sensitivity across both sample types, confirming its reliability for diagnosing BCoV infections in clinical and research settings.

In addition, a pan-coronavirus reverse transcriptase PCR (PanCoV-RT-PCR) assay was established to investigate the detection rates of BCoV in healthy and diarrheic dairy calves and compared with a BCoV-specific RT-PCR assay [36]. Results showed 55% overall BCoV positivity, with higher prevalence in diarrheic calves (64%) compared to healthy calves (46%). The study demonstrated a good consistent between the two assays (kappa = 0.68), suggesting the PanCoV-RT-PCR assay is a rapid and reliable screening tool for BCoV detection in cattle. While demonstrating potential utility for outbreak surveillance, the authors emphasized that larger-scale validation studies will carried out to confirm its broader applicability.

Calf diarrhea involves complex pathogen interactions, including bacterial, viral, and parasitic agents, with frequent mixed infections in clinically affected animals. While traditional single-gene RT-PCR remains widely used, its limitations include prolonged processing times of 4–6h, and high costs. Multiplex RT-PCR addresses these challenges by simultaneously detecting multiple pathogens in a single reaction. This approach maintains diagnostic accuracy while improving efficiency through 30–40% faster processing and reduced contamination risk from minimized sample handling. Pathogen-specific multiplex assays were developed for BCoV and other diarrheal agents, providing with rapid, comprehensive diagnostic solutions for complex infections [37,38,39,40].

Significant advances in multiplex detection assays include a triplex RT-PCR assay, which capable of simultaneously detecting three major bovine enteric pathogens in gastrointestinal tissues: BCoV, bovine rotavirus (BRV), and bovine viral diarrhea virus (BVDV) [39]. When applied to 300 buffalo calf fecal samples, this assay demonstrated a 0.9% BCoV detection rate while proving to be a rapid, sensitive, and cost-effective diagnostic solution. Similarly, an end-point multiplex RT-PCR was developed and can detect five key neonatal calf diarrhea pathogens: BRV, BCoV, *Escherichia coli K99*, *Salmonella enterica*, and *Cryptosporidium parvum* [41]. The assay achieved a detection limit of 10^−2^ dilution for BCoV-positive sample pools, and showed high analytical specificity without cross-reactivity against other common diarrheal pathogens. Comparative analysis confirmed equivalent performance to single-target RT-PCR without nonspecific amplification, establishing its utility as a cost and time-efficient tool for enhanced neonatal calf diarrhea surveillance.

RT-PCR is currently the gold standard and the most widely used assay for BCoV detection. However, the high frequency of recombination in coronaviruses drives viral evolution and poses a challenge to molecular detection. This was highlighted by the recent emergence of a novel BCoV variant, which contained a 12-nucleotide bovine gene insertion in the receptor-binding domain of a naturally recombinant HE gene. Consequently, continuous primer and probe updates are necessary. To ensure detection accuracy across diverse variants, it is critical to target highly conserved genomic regions, as assays binding to variable regions risk reduced sensitivity or false-negative results due to sequence mismatches.

### 3.2. Quantitative Real-Time PCR (qRT-PCR)

qRT-PCR offers higher specificity and sensitivity compared to conventional RT-PCR, as well as quantitative capability. This technique combines viral nucleic acid amplification and detection into a single process through continuous monitoring. The qRT-PCR workflow involves three key steps: First, reverse transcription converts BCoV genomic RNA into complementary DNA (cDNA). Second, specific cDNA regions are amplified through thermal cycling. Finally, amplification progress is monitored in real-time using either fluorescent dyes (SYBR Green) or sequence-specific probes (TaqMan) [42]. The TaqMan system achieves higher diagnostic specificity and sensitivity for BCoV detection than SYBR Green, owing to its distinct detection mechanism. During annealing, a probe labeled with both a reporter dye and a quencher hybridizes specifically to an internal target sequence during the annealing step. The 5′-3′ exonuclease activity of Taq polymerase then cleaves this hybridized probe, separating the reporter dye from quencher and generating a fluorescent signal proportional to amplified product. In contrast, SYBR Green binds nonspecifically to all double-stranded DNA, including non-target amplifications, which can lead to false-positive signals.

A previous report showed a SYBR Green I real-time RT-PCR assay targeting conserved regions of the BCoV N gene was developed to detect and quantify the virus [43]. This assay employs bovine glyceraldehyde-3-phosphate dehydrogenase (GAPDH) RNA as an internal control, achieving a detection limit of 10^3^ plasmid copies per reaction. Melting-curve analysis demonstrates high analytical specificity, showing no cross-reactivity with negative controls or structurally related coronaviruses. When validated against conventional RT-PCR using 103 clinical samples, the assay demonstrated 100% concordance. The assay detected three additional fecal samples and two nasal swabs that were negative by gel electrophoresis, confirming enhanced sensitivity. These performance characteristics establish its utility as a high-throughput diagnostic tool for BCoV surveillance and pathogenesis research.

Similarly, a TaqMan-based real-time RT-PCR assay was developed for detecting BCoV RNA in cattle clinical samples [44]. The assay utilizes primers and a 6-carboxyfluorescein (FAM)-labeled probe targeting the BCoV M gene. It showed high specificity for bovine-like coronaviruses and no cross-reactivity with other prevalent bovine viral pathogens. Additionally, the assay achieved a detection limit of 20 BCoV RNA copies per reaction, representing a 10-fold increase in sensitivity compared to conventional RT-PCR. Applied to 220 clinical specimens, the TaqMan assay demonstrated enhanced sensitivity compared to conventional RT-PCR. It detected BCoV in 49 rectal, 60 nasal, and 37 ocular samples, whereas conventional RT-PCR detected 43, 54, and 34 positives in these sample types, respectively. The assay’s rapidity, high throughput, and enhanced sensitivity make this qRT-PCR a standardized surveillance tool for BCoV in both enteric and respiratory infections.

Given its critical role in bovine diarrheal disease, BCoV was increasingly incorporated into multiplex qRT-PCR assays targeting enteric pathogens [45,46,47]. A representative one-step multiplex qRT-PCR enabled simultaneous detection of three major neonatal calf diarrhea pathogens: BRV, BCoV, and enterotoxigenic *Escherichia coli K99+* [48]. This assay achieved a detection limit of 10 copies/μL for all targets while maintaining high specificity against non-target enteric pathogens. Building on this foundation, a later-developed one-step multiplex qRT-PCR assay incorporated *Cryptosporidium parvum* detection [49]. The assay achieved detection limits of 2.3 × 10^1^–4.5 × 10^2^ copies/μL for each pathogen while maintaining high specificity without cross-reactivity. Clinical performance exceeded 90% diagnostic sensitivity and specificity for all targets, with near-perfect agreement (kappa > 0.9) versus commercial TOROIVD^®^ Gene Test Tube (TOROIVD^®^, Shanghai, China). In addition, A high-throughput multiplex qRT-PCR was subsequently established for simultaneous detection of six enteric pathogens: BCoV, BRV, BVDV, bovine enterovirus (BEV), bovine toro virus (BToV), and bovine norovirus(BNoV) [50]. This assay demonstrated high sensitivity, with detection limits of 1.91~96.0 copies/μL for each pathogen. Clinical validation using 295 diarrheic calf fecal or anal swab samples confirmed the assay’s utility for large-scale epidemiological surveillance.

Additionally, due to its role in bovine respiratory disease (BRD), BCoV is also included in multiplex qRT-PCR assays designed for respiratory pathogen detection [51,52,53]. A triplex probe-based qRT-PCR assay was established and used to simultaneously detect BCoV, bovine parvovirus (BPV), and bovine parainfluenza virus (BPIV) [51]. This assay achieved detection limits of 2.0 × 10^2^ RNA copies/μL for BPV and BCoV, and 2.0 × 10^1^ copies/μL for BPIV. This represents a 1000-fold improvement in sensitivity compared to conventional RT-PCR. Clinically validated, this assay enables efficient monitoring and differential diagnosis of co-infections with other respiratory pathogens. As well, a previous study evaluated the commercially available multiplex qRT-PCR kit(Pneumo4V), which simultaneously detect five BRD-associated viruses: BCoV, BVDV, BPIV-3,BRSVand bovine herpesvirus-1 (BoHV-1) [52]. The assay demonstrated high sensitivity with detection limits of 10–50 nucleic acid copies per reaction for all targets. This performance was further evidenced by a high agreement (0.71–0.90) with certified diagnostic qPCR assays, substantiating the kit’s status as a validated commercial product. This multiplex platform provides a reliable solution for simultaneous pathogen identification in BRD surveillance and management.

While multiplex qRT-PCR represents a powerful molecular diagnostic tool, its application presents several technical challenges. The simultaneous use of multiple primer-probe sets increases the risk of non-specific amplification, necessitating meticulous design of highly specific primers and probes tailored to target viral sequences. Furthermore, diagnostic sensitivity can be reduced during early infection due to low viral RNA loads, potentially generating false-negative results. The technique’s widespread implementation is also constrained by its dependence on sophisticated instrumentation and the need for specialized technical expertise, factors that limit its scalability, particularly in field settings.

### 3.3. Droplet Digital PCR (ddPCR)

Droplet Digital PCR (ddPCR), first developed in 1999 [54], partitions nucleic acid molecules into numerous individual reactions for amplification and quantifies targets by counting positive partitions [55]. Unlike RT-PCR, ddPCR eliminates the need for standard curves, exhibits enhanced tolerance to PCR inhibitors, and provides absolute quantification via Poisson statistics. This makes it highly sensitive and precise for low-abundance nucleic acid detection. Consequently, ddPCR is particularly valuable for detecting low-level BCoV infections in both clinical and experimental settings [56,57,58,59].

Recent advancements in ddPCR have significantly improved the detection and characterization of bovine enteric and respiratory pathogens. Comparative studies confirm ddPCR’s enhanced analytical sensitivity over conventional qRT-PCR in experimental bovine coronavirus infections [57]. While qRT-PCR reliably detected BCoV RNA in nasal and fecal samples, it failed to detect viremia. In contrast, ddPCR resolved ultra-low-level viremia as low as 0.048 copies/μL of the viral N gene in two orally inoculated calves, highlighting its utility for studying early-stage or subclinical BCoV pathogenesis.

A recent report showed a multiplex ddPCR assay was developed subsequently for simultaneously detecting of BCoV, BRV, and BEV in clinical samples [56]. This assay achieved higher sensitivity of 1 copy/μL for BCoV, representing a 1000-fold improvement than qRT-PCR, while maintaining high specificity and diagnostic accuracy. The platform’s ability to discriminate single versus mixed infections with minimal sample input establishes it as a highly sensitive diagnostic tool for bovine diarrheal diseases, particularly in complex co-infection where conventional methods lack resolution.

ddPCR offers higher sensitivity compared to qRT-PCR, enabling reliable detection of low viral loads and reducing false-negative results, making it as a valuable diagnostic tool. However, clinical implementation remains limited by technical complexity, dependence on specialized operators, and requirements for advanced instruments. These constraints restrict ddPCR primarily to centralized laboratories, preventing its application in resource-limited settings.

### 3.4. Next-Generation Sequencing (NGS)

NGS assay has changed how we study viruses like BCoV. By directly analyzing genetic material from clinical samples, NGS provides a powerful method to detect and characterize pathogens without needing prior knowledge of their identity. A typical NGS workflow involves extracting viral RNA from clinical samples such as fecal or respiratory secretions, constructing libraries for sequencing, and performing bioinformatic comparisons against reference databases to resolve genomic homology and evolutionary relationships. For more focused applications, targeted NGS employs primers to amplify specific genomic segments, thereby enriching viral content to aid in highly sensitive detection and sequencing. This powerful capabilities make NGS indispensable for investigating outbreaks, understanding viral transmission dynamics, and tracking viral evolution.

NGS has become a powerful tool for studying BCoV, offering unique capabilities for tracking viral diversity and evolution. Recent research utilized NGS to characterize BCoV genomic diversity and evolution in Chinese cattle, particularly examining its association with diarrhea [60]. Their analysis of clinical samples from both symptomatic and asymptomatic animals across five regions yielded nearly complete genomes for six BCoV strains. Validation through RT-PCR and Sanger sequencing showed complete agreement with NGS results, though 3′ and 5′ terminal regions remained unresolved due to technical limitations. This work shows NGS is a powerful tool for viral genome analysis, providing valuable genomic references for tracking BCoV’s evolution.

Importantly, NGS has clarified key virus–host interactions while revealing molecular determinants of BCoV tissue tropism. An investigative approach employing NGS analyzed host factors in BCoV infection, comparing bovine epithelial cells infected with enteric (BCoV/Ent) versus respiratory (BCoV/Resp) strains [61]. Post-infection transcriptomic profiling identified differentially expressed host genes potentially involved in viral entry and replication mechanisms. Subsequent qRT-PCR validation using both infected and control lung tissue samples confirmed the reliability of the NGS findings. This NGS-based approach not only identified critical host–pathogen interactions but also demonstrated the utility of high-throughput sequencing in uncovering molecular mechanisms underlying viral tropism.

In summary, NGS provides superior insights into genomic diversity and subtle genetic variations, significantly advancing our understanding of BCoV evolution and pathogenesis. This technology offers key advantages, including high-throughput detection, high sensitivity, and the ability to characterize full-length viral genomes in a single sequencing run. However, its reliance on specialized equipment, substantial computational resources, and technical expertise, along with higher costs and longer processing times compared to conventional methods like RT-PCR, currently limits widespread usein routine clinical diagnostics.

### 3.5. Isothermal Amplification

Isothermal amplification offers a practical alternative to conventional PCR by maintaining high detection sensitivity while eliminating the need for thermal cycling. These techniques, including recombinase polymerase amplification, recombinase-aided amplification, and reverse transcription loop-mediated isothermal amplification, enable rapid nucleic acid amplification at constant temperatures, generating results within 5–30 min. These characteristics make them particularly valuable for field diagnostics. Notably, these approaches have been successfully applied to BCoV detection, with a growing body of evidence validating their efficacy for rapid on-site diagnosis.

#### 3.5.1. Reverse Transcription Loop-Mediated Isothermal Amplification (RT-LAMP)

RT-LAMP has emerged as one of the most widely adopted isothermal amplification methods since its development in 2000. This technique utilizes 4–6 specialized primers and Bst DNA polymerase to enable highly specific nucleic acid amplification at a constant temperature of 60–65 °C, eliminating the need for thermal cycling. Through its unique strand displacement mechanism, RT-LAMP can generate up to 10^9^ DNA copies within 30–60 min, resulting in characteristic loop-structured amplicons. The method offers multiple endpoint detection options, including visual colorimetric changes, turbidity measurements, and fluorescence readouts. Currently, RT-LAMP has demonstrated particular effectiveness in BCoV detection and has well-established applications in diagnosing calf diarrhea outbreaks.

An RT-LAMP assay targeting the BCoV N gene was also developed and employed for SYBR Green I visualization as well as gel electrophoresis [62]. The assay demonstrated high specificity for BCoV, showing no cross-reactivity with other pathogens, and achieved high sensitivity, with 98.2% agreement with nested RT-PCR. Notably, the test’s performance varies by sample type, with a limit of detection of 10^2^ copies per reaction in fecal samples and 10 copies per reaction in purified plasmid DNA, highlighting the strong inhibitor tolerance in the assay. Clinical evaluation using 418 samples from Xinjiang province confirmed its reliability as a simple, rapid, and accurate diagnostic tool for field applications. These findings validate RT-LAMP’s potential for BCoV surveillance, although considerations regarding sample type remain crucial for optimal implementation.

Unlike PCR requiring thermal cycling, RT-LAMP operates at constant temperature, allowing use with basic equipment. This method maintains high sensitivity and tolerates impure samples while delivering results under 60 min. Although primer design remains challenging, RT-LAMP’s simple instrumentation and strong inhibitor tolerance make it an ideal PCR alternative for field testing, especially in resource-limited settings.

#### 3.5.2. Recombinase Polymerase Amplification (RPA)

RPA was first developed in 2006 and has become a widely adopted isothermal nucleic acid amplification technique for pathogen detection [63,64,65,66]. The RPA system comprises three key components: a recombinase, a strand-displacing polymerase, and a single-stranded DNA-binding protein (SSB). This innovative technology operates at moderate temperatures (37–42 °C) without thermal cycling and can generate detectable amplification products within 30 min using simple incubation devices such as a water bath or heating block [67]. Recombinase-aided amplification (RAA) is closely related to RPA. The primary difference lies in the source of the recombinase: RAA utilizes enzymes derived from bacteria or fungi, whereas RPA employs theT4 bacteriophage-derived UvsX recombinase [68].

**Figure 2:**
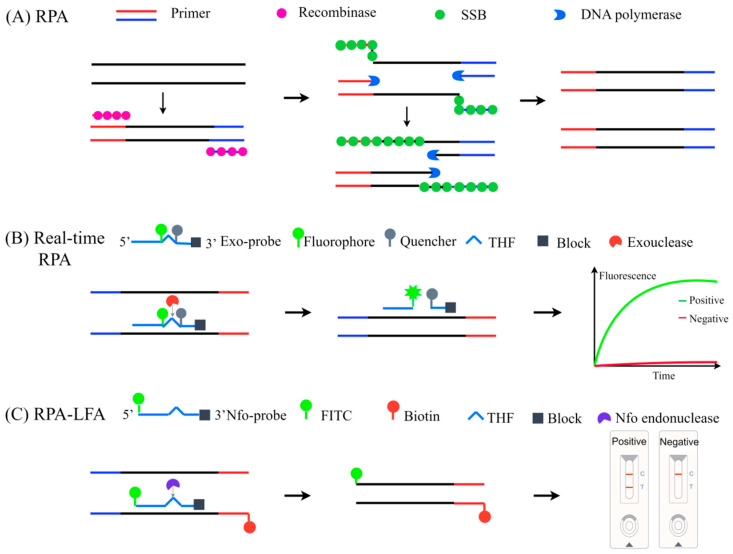
Schematic representation of RPA and detection methods. (**A**) RPA principle: Primer-recombinase complexes enable exponential isothermal amplification. (**B**) Real-time RPA: Exonuclease cleavage at a basic sites separates fluorophore/quencher pairs for real-time fluorescence. (**C**) RPA-LFA: Biotinylated primers and FITC-probes generate dual-labeled amplicons; blocker cleavage enables extension, with detection via lateral flow strip.

The reaction mechanism model of RPA is shown in Figure 2. First, the recombinase UvsX binds to primers and ATP to form a complex, which then scans for homologous sequences in the double-stranded DNA. Upon recognizing homologous sequences, the complex localizes to the template DNA and facilitates strand displacement. Single-stranded DNA-binding protein stabilizes the resulting displaced DNA structure. After dissociation of the recombinase, the 3′ end of the primer is exposed. DNA polymerase binds to the 3′ end of the primer to catalyze DNA extension and synthesize new DNA strands. Through iterative cycles of this process, the target DNA is amplified exponentially from minute initial amounts, reaching detectable levels within minutes [69]. Finally, amplicon detection methods include gel electrophoresis, real-time fluorescence detection, and lateral flow strip detection [70,71,72,73].

Real-time fluorescence detection employs a dual-labeled probe containing fluorophore and quencher groups, designed with an internal tetrahydrofuran (THF) a basic site substituting a target-specific nucleotide. When intact, fluorescence quenching occurs due to fluorophore-quencher proximity. The probe’s 3′-terminus is modified with a C3-spacer or phosphate group to prevent polymerase extension. During amplification, probe hybridization to complementary targets enables Exonuclease III cleavage at the abasic site, separating fluorophore from quencher and generating detectable fluorescence signals.

A real-time fluorescent RPA assay was developed and targeted the BCoV N gene for detection in bovine fecal samples and nasal swabs [74]. The assay demonstrated high sensitivity, detecting as few as 19 RNA target molecules per reaction, and demonstrated excellent specificity, showing no cross-reactivity with other bovine respiratory or enteric viruses. Preliminary clinical validation using 30 samples (16 fecal samples and 14 nasal swabs) showed full concordance with qRT-PCR while achieving rapid results within 10–20 min at 37–42 °C. The assay maintains high diagnostic accuracy with rapid turnaround, while its compatibility with handheld fluorescence readers enables deployment in both laboratory and field-based BCoV diagnostics.

Additionally, RT-RAA works through two diagnostic formats: real-time fluorescence and lateral flow strip detection [75]. Both formats demonstrated high specificity with no cross-reactivity against related viruses and significantly enhanced sensitivity compared to conventional RT-PCR, achieving detection limits of 1.46 × 10^1^ copies/μL for real-time RT-RAA and 1.46 × 10^2^ copies/μL for lateral flow strip-based RT-RAA. Evaluation using 242 clinical samples revealed that real-time RT-RAA exhibited sensitivity comparable to RT-qPCR, while lateral flow strip-based RT-RAA format showed greater sensitivity than conventional RT-PCR. These advances represent field-deployable diagnostics combining isothermal amplification simplicity with laboratory-grade accuracy. Multiplex RAA has also demonstrated superior efficiency over single plex RAA, enabling the simultaneous detection of multiple different viruses in a single reaction [76].

RPA offers distinct advantages for BCoV detection compared to conventional methods. Unlike RT-qPCR, which requires sophisticated thermocyclers, and RT-LAMP, which utilizes multiple primers (typically 4–6) and operates at higher temperatures (60–65 °C), RPA/RAA requires only two target-specific primers, operates at moderate temperatures (37–42 °C), and delivers results within 10–30 min [77]. Moreover, these methods require minimal equipment and enable visual readouts, rendering them particularly suitable for field applications [71].

### 3.6. CRISPR/Cas13a Diagnostics

Clustered regularly interspaced short palindromic repeats (CRISPR) systems, originally discovered in 1987, have evolved from a bacterial adaptive immune mechanism into a powerful platform for molecular diagnostics [78]. CRISPR systems are broadly classified into two major classes (1 and 2), each comprising multiple types defined by distinct Cas protein structures and sequence features [79]. Among these, the Class 2 Type VI effector protein Cas13a has proven particularly valuable for diagnostic applications owing to its operational simplicity [80].

CRISPR/Cas13a systems detect target RNA through a two-step mechanism: first, sequence-specific recognition and binding directed by a CRISPR RNA (crRNA) guide; second, activation of its nonspecific collateral RNase activity upon target binding. Specifically, after crRNA-guided binding to complementary single-stranded RNA (ssRNA), the activated Cas13a releases its non-specific RNase activity, cleaving nearby ssRNAmolecules indiscriminately, generating amplified signals detectable via fluorescence or lateral flow strips [81]. This approach demonstrates significant potential for RNA virus detection including BCoV.

**Figure 3:**
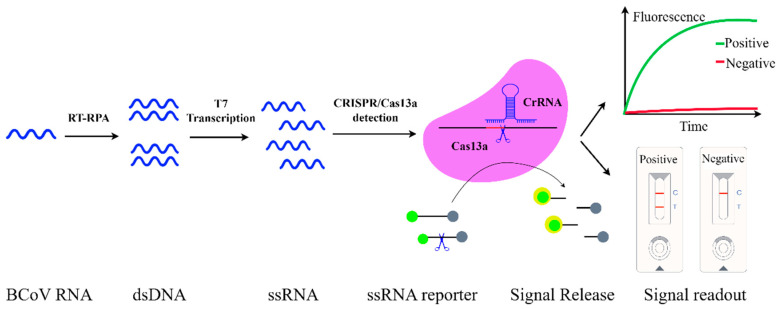
Schematic representation of CRISPR/Cas13adiagnosticsof BCoV. BCoV RNA is amplified by reverse transcription RPA (RT-RPA), followed by T7 transcription of dsDNA amplicons into RNA. The Cas13a/crRNA complex binds this RNA, activating trans-cleavage of an added ssRNA reporter. Detection occurs through fluorescence or LFA readouts.

To enhance diagnostic sensitivity and specificity, CRISPR/Cas13a systems are commonly integrated with isothermal amplification methods such as RPA or RAA. These techniques amplify target nucleic acids at constant temperatures, generating sufficient substrate for sequence-specific detection via Cas13a collateral cleavage (Figure 3). An integrated diagnostic assay was established and executed RT-RAA coupled with CRISPR/Cas13a-mediated fluorescent detection, targeting the highly conserved BCoV N gene [82]. Validation using clinical specimens from beef cattle and yaks demonstrated a detection sensitivity of 1.73 copies/μL and high specificity. BCoV was detected in 58.3% of samples, substantially exceeding the 2.4% detection rate of conventional RT-qPCR, with all positive results confirmed by Sanger sequencing. Critically, the system enabled reliable detection across diverse matrices including respiratory secretions and fecal specimens, overcoming limitations in field surveillance applications while maintaining consistent performance across sample types.

CRISPR/Cas13adiagnostics represent a transformative advancement for RNA virus detection, achieving high laboratory-grade sensitivity and 100% specificity within 40 min through programmable RNA targeting and collateral cleavage activity. As demonstrated in BCoV surveillance applications, these systems offer flexible readout modalities including fluorescence and lateral flow strips, which make them particularly valuable for field-deployable diagnostics that maintain high accuracy across variable sample conditions [83].

## 4. Immunological Methods for Antigen and Antibody Detection

Immunological methods detect BCoV through antigen–antibody interactions, utilizing antigen tests to identify viral proteins and antibody tests to measure host immune responses. These approaches play a critical role in seroprevalence studies and vaccine efficacy evaluations. Among them, lateral flow assay and enzyme-linked immunosorbent assay are the most widely adopted due to their simplicity and reliability.

### 4.1. Enzyme-Linked Immunosorbent Assay (ELISA)

**Figure 4:**
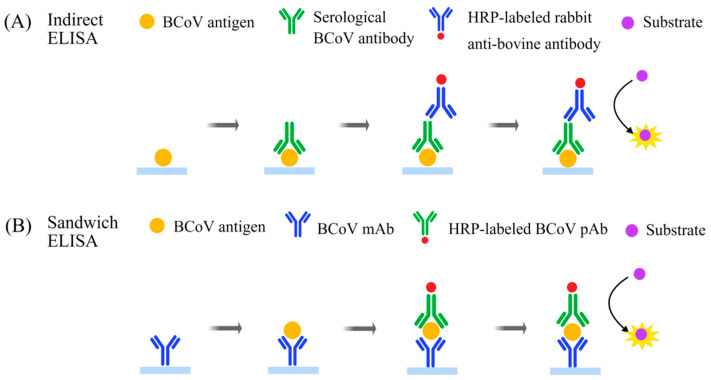
Schematic presentation of two ELISA methods. (**A**) Indirect ELISA for detecting BCoV antibody in serum/milk; (**B**) Sandwich ELISA for detecting BCoV antigen in fecal/nasal samples.

ELISA is a high-throughput quantitative immunoassay that detects antigens or antibodies using enzyme-conjugated antibodies. This technique offers high sensitivity and specificity for pathogen detection [84]. Its core principle involves using specific capture antigens or antibodies to bind selectively to target antibodies or antigenic sites, respectively. Following this specific binding, a reporter system utilizing enzyme-conjugated antibodies generates a measurable signal, thereby identifying positive samples. For BCoV, ELISA serves as a primary diagnostic tool for viral antigen detection and serological antibody monitoring (Figure 4).

Several studies have demonstrated the effectiveness of monoclonal antibody (mAb)-based ELISA for detecting BCoV in fecal samples. A sandwich ELISA, which using the Z3A5 monoclonal antibody against the S protein, showed enhanced sensitivity, detecting 10^4^ virus particles compared to electron microscopy (10^5^ particles) and hemagglutination assays [85]. This assay achieved 92% agreement and 96% specificity versus electron microscopy, establishing its utility for large-scale fecal surveillance. Further refinement using a cocktail of four monoclonal antibodies (two anti-S and two anti-N) in a sandwich ELISA format demonstrated 87.7% agreement with electron microscopy when testing diarrheic calf fecal samples [86]. These findings collectively support mAb-based sandwich ELISA as a reference method for BCoV detection in fecal samples.

Moreover, an innovative isotype-specific capture ELISA was developed for detection of BCoV-specific IgA and IgM antibodies in milk and serum specimens [87]. This assay demonstrated enhanced sensitivity compared to conventional indirect ELISA while maintaining high diagnostic reliability. Its capacity, which distinguishes primary BCoV infections from reinfections in bovines, were demonstrated through distinct serological profiles, with reinfected animals exhibiting sustained peak IgA concentrations in both matrices. The method’s utility is augmented by compatibility with milk sampling, enabling non-invasive monitoring of mucosal IgA and systemic IgM responses in lactating herds.

Recent reports showed a novel multiplex antibody ELISA was developed and used for BCoV surveillance through detecting bulk milk samples [88]. This assay simultaneously targets BCoV and BRSV, utilizing three recombinant proteins (A–C) as antigens. Compared to the commercial ELISA Kit (SVANOVIRBCV-Ab. SVANOVA, Uppsala, Sweden), the multiplex assay demonstrated 99.9% sensitivity and 93.7% specificity for BCoV under Bayesian modeling-optimized conditions. Large-scale validation with 720 bulk milk samples identified the multiplex ELISA’s performance, establishing it as a high-throughput population-level surveillance tool. While SVANOVIR^®^ ELISA remains prevalent in high-income countries due to its accuracy and program integration, the multiplex ELISA offers enhanced versatility through simultaneous multi-pathogen detection.

Recently, a N protein-based indirect ELISA was developed for BCoV antibody detection in serum [89]. The assay utilized CHO cell-expressed recombinant N protein as capture antigen paired with rabbit polyclonal antibodies generated against the immunogen. Through checkerboard titration optimization, critical parameters were established at an antigen concentration of 1.25 μg/mL, serum dilution of 1:200, and diagnostic cutoff value of 0.986. When validated against 58 bovine serum samples, the optimized indirect ELISA demonstrated high diagnostic performance with 100% sensitivity, 94.64% specificity, and 94.83% concordance relative to a commercial reference kit(Keshun Biotechnology, Shanghai, China). This N protein-based format provides a standardized diagnostic alternative for applications requiring rapid serological assessment of BCoV exposure.

A growing number of studies have successfully identified bovine-like coronaviruses in a variety of non-bovine ruminants, including water buffalo, camelids, goats, and giraffes [90,91,92]. Given the close antigenic relationship between BCoV and bovine-like coronaviruses, diagnostic assays validated for cattle are often applied off-label in these non-bovine ruminants. For example, a commercial ELISA Test Kit (SVANOVIR BCV-Ab. SVANOVA, Uppsala, Sweden) used in southern Italy detected a significantly lower seroprevalence in water buffalo (5.3%) than in cattle (49.2%), illustrating both this practice and potential host-specific differences in exposure or assay performance [90]. This approach is supported by demonstrated serological cross-reactivity, as evidenced by a bovine-like coronavirus isolated from a giraffe in a wild-animal park in the United States, which was detectable by a BCoV-specific indirect ELISA [93]. While the serological cross-reactivity among bovine-like coronaviruses enables broad pathogen screening, it introduces a key diagnostic limitation: a positive BCoV ELISA result may reflect infection with either BCoV or a cross-reacting bovine-like virus, potentially leading to false-positive interpretations, particularly in non-bovine species. Therefore, although BCoV ELISAs provide a practical initial surveillance tool, results should be interpreted with caution and confirmed where necessary. To address this, the same samples should be tested using specific serum neutralization tests for BCoV and bovine-like coronaviruses.

ELISA has been successfully adapted for BCoV diagnosis across diverse sample matrices, including feces, milk, and serum. These assays demonstrate consistently high sensitivity and specificity while requiring only basic laboratory equipment, enhancing their accessibility for routine diagnostics. Furthermore, their high-throughput capability enables efficient clinical diagnosis and large-scale epidemiological studies. However, the main limitation of this method is its inability to distinguish between active and past infections. Therefore, results must be interpreted with caution, as detected antibodies may not signify an active infection. In some cases, confirmation by PCR is required.

### 4.2. Serum Neutralization Test (SNT)

The SNT serves as an important tool for assessing antibody neutralizing capacity, diagnosing viral infections, and evaluating vaccine immunogenicity. It is widely applied in clinical diagnosis, vaccine development, and epidemiological research. Despite its procedural complexity, its accuracy and reproducibility have established it as a gold standard method.

Several studies have demonstrated the significant utility of the SNT across multiple domains of BCoV research. Its application in cross-species monitoring was established as early as 1985, when virus neutralization and hemagglutination inhibition assays successfully detected BCoV antibodies in water buffalo populations. It was concluded that a hemagglutinating coronavirus closely related to BCoV was circulating among buffalo populations [94]. Similar viruses were subsequently detected as bovine-like coronaviruses in a variety of non-bovine ruminants. While bovine-like coronaviruses exhibit cross-reactivity with BCoV antisera in ELISA, the SNT provides definitive confirmation through virus-specific protocols for BCoV and bovine-like coronaviruses. The method’s capacity to confirm infection by detecting functional immune responses was further validated in a recent study evaluating multiplex qPCR for active infection surveillance [53]. In vaccine development, neutralization tests provide a superior assessment of protective immunity. This was demonstrated in an early study where antibodies induced by the Mebus vaccine effectively neutralized genetically distinct field strains, validating broad vaccine coverage [95]. Another study further indicates that if significant antigenic differences are found in local variants, they should be considered for testing and developing effective BCoV vaccines [96]. Thus, the SNT is therefore indispensable to BCoV research, proving essential for diagnostic confirmation and the assessment of vaccine efficacy [97].

Despite its high diagnostic specificity, the practical implementation of the SNT is limited by several operational constraints. These include the requirement for live virus or pseudo virus systems, specialized cell culture facilities, considerable technical expertise, and an extended turnaround time of several days. Consequently, the SNT is less suitable for rapid, high-throughput field applications compared to ELISA or lateral flow assays. Its deployment is therefore best reserved for situations requiring definitive diagnostic confirmation or detailed immunological evaluation.

### 4.3. Lateral Flow Assay (LFA)

**Figure 5:**
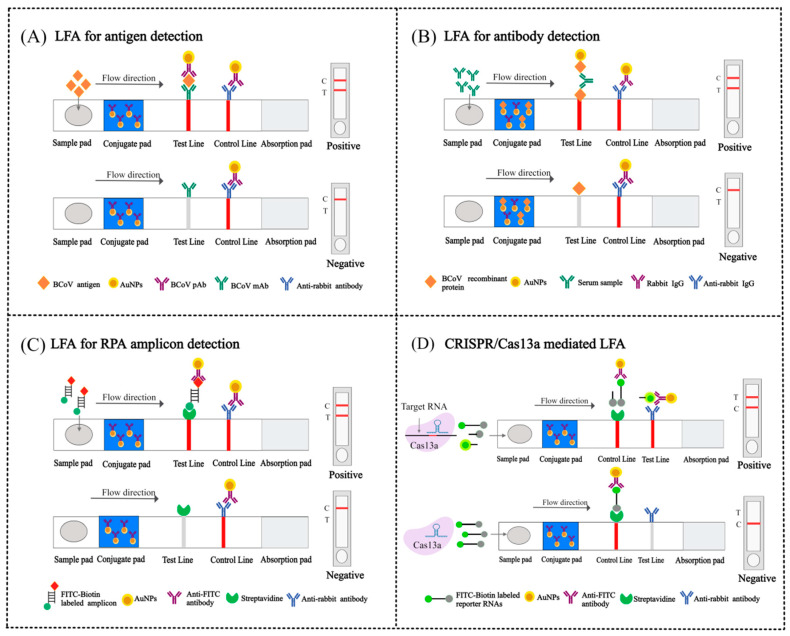
Schematic representation of AuNPs based LFA. (**A**) LFA for antigen detection: Viral antigens bind antibody-conjugated AuNPs and are captured at the test line by immobilized antibodies. (**B**) LFA for antibody detection: Serum antibodies bind antigen-conjugated AuNPs, captured at the test line by immobilized antigens, with unbound complexes binding the control line. (**C**) LFA for RPA amplicon detection: Biotin/FITC-labeled amplicons bind anti-FITC-AuNPs and are captured at the test line by streptavidin, while the control line binds antibody-AuNP complexes. (**D**) CRISPR/Cas13a mediated LFA: Target-activated Cas13 cleaves the FITC-biotin reporter; intact reporters bind streptavidin at the test line (single band), while cleaved FITC binds antibody-AuNPs captured at the test line, with the control line generating dual bands for positive results (By Figdraw).

LFA is a rapid diagnostic platform that integrates antibody–antigen binding with chromatographic separation to detect target analytes such as proteins, antibodies, or nucleic acids [98,99] (Figure 5). A standard LFA strip comprises several key components assembled on a backing plate: a sample pad, a conjugate pad, a nitrocellulose membrane pre-printed with test and control lines, and an absorbent pad [100,101].

Commonly, LFA is employed for detecting either antigens or antibodies. In antigen detection, viral antigens present in the sample first bind to antibody-conjugated gold nanoparticles (AuNPs) on the conjugate pad. This complex then migrates along the strip and is captured at the test line by immobilized antibodies specific to the target antigen (Figure 5A). Conversely, for antibody detection, antibodies within a serum sample bind to antigen-conjugated AuNPs. These complexes are subsequently captured at the test line by immobilized antigens. Unbound complexes from either format are captured at the control line by secondary antibodies, confirming the test’s validity (Figure 5B). This entire process can be completed within 10 min [102].

Over the past few decades, LFA has been developed as one of the most widely used diagnostic tools. It provides a practical alternative to laboratory-based methods, proving particularly valuable in resource-limited settings [103,104]. Although traditional LFAwas often limited by low sensitivity, recent advancements integrating nanotechnology and molecular techniques like RT-LAMP, CRISPR systems, and RPA have largely enhanced their capabilities. Modern LFA now achieve field adaptability while maintaining laboratory-grade accuracy. This progress has solidified their essential role in sensitive and specific detection applications, including BCoV diagnosis.

#### 4.3.1. RT-LAMP-LFA

A previous study showed that an established integrated duplex RT-LAMP-LFA platform on a single microfluidic chip enables simultaneous detection and differentiation of BCoV and Influenza A/X-31 (InfA), achieving limit of 100 copies/reaction and 10 copies/reaction, respectively [105]. This system employs bifunctional primers labeled with biotin (BCoV) and digoxigenin (InfA), enabling concurrent target amplification and discrimination via lateral flow strips. Amplified products bind to strip-specific capture zones through ligand–receptor interactions (streptavidin-biotin and anti-digoxigenin-digoxigenin), generating visually interpretable results within 20–30 min. The device pre-integrates primers, reagents, and pre-embedded lateral flow strips, requiring only manual sample activation while maintaining >7-day result stability. By combining RT-LAMP’s analytical sensitivity with LFA’s field portability, this integrated approach represents a scalable, field-deployable solution for multiplexed pathogen surveillance.

#### 4.3.2. RPA-LFA

RPA coupled with LFA (RPA-LFA) provides a rapid diagnostic platform for BCoV detection [106]. This method employs dual-labeled molecular tags: a probe containing an internal THF site with 5′-FITC/FAM, paired with a 5′-biotinylated reverse primer. During isothermal amplification, target-specific probe hybridization triggers enzymatic cleavage at the THF site by endonuclease, followed by DNA polymerase extension to generate double-stranded amplicons dually labeled with FITC and biotin [107]. For detection, these amplicons bind to anti-FITC antibody-conjugated AuNPs on the lateral flow strip. Under capillary flow, streptavidin at the test line captures the biotin tag while anti-rabbit IgG antibodies at the control line immobilize excess AuNP complexes [108] (Figure 5C).

Complementing these approaches, a multienzyme isothermal rapid amplification coupled with LFA (MIRA-LFA) platform was developed to target a highly conserved region of the BCoV N gene [106]. The method employs biotin- and FAM-labeled primers to generate dual-tagged amplicons, which complex with gold-conjugated anti-FAM antibodies. During lateral flow detection, the test line captures target complexes via biotin-streptavidin interaction, while the control line immobilizes excess complexes using species-specific anti-rabbit antibodies. Validation studies established a detection limit of 100 plasmid copies, demonstrating high analytical specificity and near-perfect clinical agreement (kappa = 0.982) with RT-qPCR when evaluated against 192 bovine clinical specimens, including nasal swabs and fecal samples. Requiring no specialized equipment and capable of processing diverse sample types, this visually interpreted platform demonstrates significant utility for on-site BCoV surveillance in field settings.

RPA-LFA technology has advanced significantly, characterized by compact instrumentation and streamlined protocols that minimize operator expertise requirements. These attributes establish RPA-LFA as a promising field-deployable alternative to conventional PCR, requiring only basic equipment such as centrifuges and water baths. Nevertheless, further optimization remains essential for implementation in resource-limited settings.

#### 4.3.3. CRISPR/Cas13a-Based LFA

While RPA-LFA offers a cost-effective, rapid, and user-friendly platform for BCoV detection, it faces challenges such as relatively low sensitivity and susceptibility to false-positive results, which limit diagnostic reliability [109]. To overcome these limitations, researchers are creating innovative signal amplification methods. Particularly promising is the integration of CRISPR systems following RPA. In the CRISPR/Cas13a-LFA workflow (Figure 5D), target-activated Cas13 cleaves FITC-biotin reporters, intact reporters bind streptavidin at the test line yielding a single band for negatives, whereas cleaved FITC fragments bind antibody-AuNPs captured at the test line, with the control line generating dual bands for positive validation. This integrated approach significantly enhances the sensitivity and specificity of LFA detection, effectively overcoming the limitations of conventional methods [110,111].

The SHERLOCK (Specific High-sensitivity Enzymatic Reporter unLOCKing) platform was developed through synergistic integration of RPA with CRISPR/Cas13a detection [80]. This method combines RPA’s exponential nucleic acid amplification with CRISPR/Cas13a’s sequence-specific recognition, achieving high analytical sensitivity with detection limits of ≤1 copy/μL and eliminating false positives from non-specific amplification products. Diagnostic applicability is further improved through lateral flow detection, enabling equipment-free visual interpretation of results.

Building on this foundation, the SHERLOCK platform was established and used specifically for detecting BCoV in diarrheic calves [83]. Their diagnostic system integrates three core technologies: RT-RPA for rapid isothermal amplification of target RNA, CRISPR/Cas13a for sequence-specific nucleic acid recognition, and LFA for visual result interpretation. This approach demonstrated high specificity and sensitivity, detecting as few as 10 RNA copies/μL within 40 min, representing a major advancement for rapid BCoV diagnostics. Although the current protocol requires an RNA extraction step from clinical samples, the assay’s impressive performance highlights its significant potential for field-deployable BCoV diagnostics. Further development of extraction-free protocols would enhance utility as a surveillance tool for livestock agriculture.

The integration of CRISPR/Cas13a-enhanced detection with lateral flow readout reduces non-specific binding while enhancing sensitivity 10- to 100-fold relative to RPA-LFA. This combined approach leverages RPA’s procedural simplicity and CRISPR’s molecular discrimination capacity, establishing a transformative framework for field diagnostics with improved detection limits and specificity [83,112]. Despite these advantages, current dependencies on RNA extraction from complex matrices and specialized equipment hinder widespread adoption in resource-limited settings [113,114]. Future efforts must therefore prioritize developing fully integrated platforms that consolidate sample processing, nucleic acid amplification, and CRISPR-based detection into unified systems to bridge diagnostic robustness and field applicability.

## 5. Future Outlook

The future of BCoV diagnostics is increasingly shifting toward field-deployable platforms, driven by the need for rapid, accessible, and cost-effective solutions across diverse settings. Table 1 presents a comparison of the sensitivity, specificity, and detection limit of various detection techniques. Conventional approaches, including molecular methods such as RT-PCR/qRT-PCR and immunological assays like ELISA and neutralization tests, remain the main diagnostic methods for BCoV. Nevertheless, they are constrained by laboratory dependency as well as the necessary balance between speed and sensitivity. These limitations have promoted the development of innovative methods, including isothermal amplification, CRISPR/Cas systems and LFA. However, such cutting-edge innovations remain largely confined to the research domain. Although proof-of-concept studies demonstrate considerable promise, their validation has so far been restricted to controlled laboratory conditions and limited clinical sample sets, and they have not yet been widely adopted as standardized commercial diagnostic kits suitable for routine application.

The integration of microfluidics and LFA into field-deployable platforms is gaining traction for BCoV detection. Microfluidic systems miniaturize and automate nucleic acid extraction, amplification, and detection, enabling portable, user-friendly devices with minimal reagent consumption [115]. For instance, RT-LAMP-integrated microfluidic systems have demonstrated promise through duplex assays detecting BCoV at concentrations of 100 copies/reaction within 60 min using self-contained cartridges [105]. Although LFA provides rapid, equipment-free results ideal for field deployment, its qualitative readouts and moderate sensitivity (typically 60–90%) compared to gold-standard molecular methods remain limitations [85,116]. These constraints particularly impact detection of low viral loads (<10^3^ copies/μL) and early-stage infections. To address these constraints, CRISPR systems integrated with isothermal amplification and LFA represent a breakthrough, achieving detection limits as low as 10 viral copies per reaction within 40 min [83]. This approach leverages RPA’s amplification efficiency and CRISPR’s target precision, positioning RPA-CRISPR-LFA as a highly promising next-generation point-of-care BCoV diagnostics [117].

Nevertheless, the practical implementation of point-of-care diagnostics for BCoV faces multiple challenges. Sample preparation remains a critical bottleneck, particularly due to matrix effects in fecal samples where inhibitors may drastically reduce sensitivity [63]. Extraction-free protocols using direct lysis buffers offer a promising alternative to conventional methods. However, they require further optimization to achieve integration of compatible sample processing, efficient nucleic acid amplification, and downstream detection into a unified microfluidic platform [118]. Given that BCoV frequently co-infection with pathogens such as BRV and BVDV, multiplex detection capability should be considered in diagnostic development. Emerging innovation like CRISPR-based multiplex detection shows particular promise for simultaneous differential detection of these epidemiologically linked pathogens [119]. Furthermore, The integration of microfluidic sample processing with LFA readouts, coupled with digital tools and artificial intelligence, should be taken into consideration to enhance accessibility and accuracy in resource-limited settings [115,120].

As these technologies mature, point-of-care diagnostics will become the frontline solution for BCoV, providing veterinarians with timely insights for disease control. The ideal diagnostic platform must simultaneously provide sensitive, specific and quantitative results with operational simplicity appropriate for diverse field conditions. Microfluidic systems that integrate isothermal amplification with LFA detection represent a particularly promising approach, offering a transformative solution that bridges the critical gap between conventional laboratory testing and reliable field diagnostics in resource-limited settings.

## 6. Conclusions

The diagnosis of BCoV remains critical for bovine disease management due to its substantial economic impact on dairy and beef production systems. While molecular assays provide high sensitivity and specificity, their reliance on specialized equipment and trained personnel limits widespread adoption. In contrast, serological methods, despite greater accessibility and cost-effectiveness, exhibit lower diagnostic resolution than nucleic acid-based techniques. Emerging technologies such as NGS and CRISPR-based systems demonstrate transformative potential to revolutionize BCoV diagnostics. NGS enables high-resolution viral evolution tracking, while CRISPR-based systems like integrated RPA-CRISPR-LFA facilitate instrument-free detection within 45 min at single-copy sensitivity. These capabilities highlight their significant potential for field applications. Despite these advances, multiple challenges hinder the development of point-of-care BCoV diagnostics. Nevertheless, ongoing innovations integrating artificial intelligence and digital tools with microfluidic systems are set to establish point-of-care diagnostics as the future surveillance standard, offering laboratory-accurate, real-time pen-side solutions for effective BCoV control.

## Figures and Tables

**Table 1:** Comparison of Diagnostic Methods for BCoV Detection.

Detection Method	Target	Detection Time	Limit of Detection	Technical Advantages and Limitations	Applicability	Reference
RT-PCR	N gene	4–6 h	200 RNA copies/reaction	High standardization; Multiplex capability; Thermal cycler required	Laboratory	[34]
qRT-PCR	M gene	2–3 h	20 RNA Copies/reaction	High standardization; Multiplex capability; Thermal cycle required	Laboratory	[44]
ddPCR	N gene	3–4 h	0.048 RNA copies/μL	Reference sensitivity; Absolute quantification; High reagent cost	Laboratory	[57]
RT-LAMP	N gene	50 min	100 RNA copies/reaction	Inhibitor tolerance; 4–6 primers per target; Constant temperature	Laboratory	[62]
Real-time RPA	N gene	10–30 min	19 RNA copies/reaction	Rapid amplification (37–42 °C); No thermal cycling; non-specificity amplification risk	laboratory	[74]
RPA-CRISPR- Fluorescence Assay	N gene	30 min	1.73 RNA copies/μL	Enhanced Sensitivity and Specificity; High reagent cost	Laboratory	[82]
RT-LAMP-LFA	N gene	50–60 min	100 RNA copies/reaction	Minimal equipment; Lyophilized reagents; Field-compatible	Field	[105]
RPA-LFA	N gene	40 min	146 RNA copies/μL	Rapid field protocol; Ambient-temperature storage	Field	[75]
RPA-CRISPR-LFA	M gene	40 min	10 RNA copies/μL	High sensitivity; Visual readout; Matrix interference resistance	Field	[83]
ELISA	N protein	2–4 h	1.25 μg/mL	High-throughput screening; Serum/fecal specimen compatibility	Laboratory	[87]
